# Supplementary material for: Systematic mapping of insertion-tolerant regions enables capsid engineering of an infectious RNA phage
Source: mBio. 2026 Aug 10;17(9):e01639-26. doi: 10.1128/mbio.01639-26 (PMC13556216; doi:10.1128/mbio.01639-26)
Supplement: Supplementary file 1 [file mbio.01639-26-s0001.pdf]

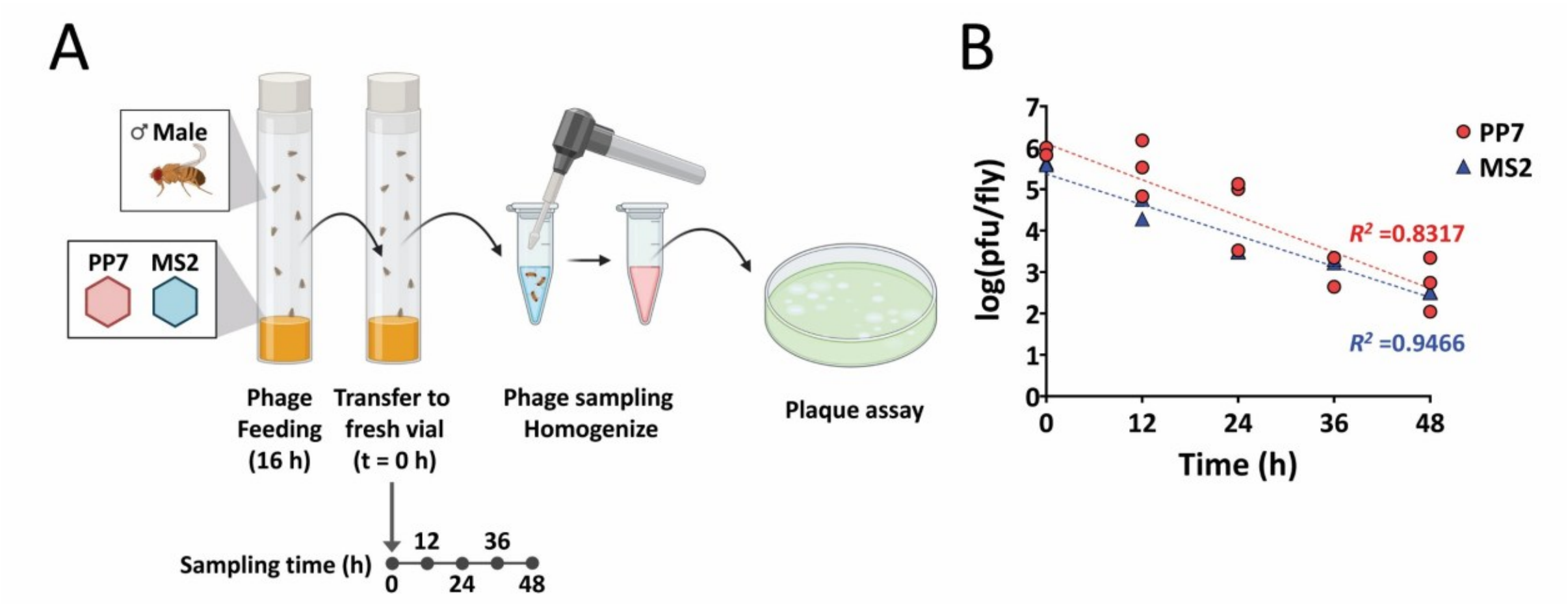

Figure S1. Stability of PP7 and MS2 phages in *Drosophila*, related to Fig. 2

A. Experimental scheme for assessing phage stability in the *Drosophila* feeding model. Male flies were fed in the medium overlaid with either PP7 or MS2 phages (~10^{10} pfu) for 16 h and then transferred to fresh, phage-free media. Three flies were collected and homogenized at every 12 h for 48 h. The resulting homogenates were subjected to plaque assay to quantify the plaque forming units (pfu) from the homogenates.

B. In vivo stability of PP7 and MS2 phages. The pfu values determined from the homogenates in **A** are calculated as log(pfu/fly) over time. The slopes reflect the in vivo stability of the fed phages, with the R^2 values indicated for both phages. The stability of both phages were not statistically significant based on ANCOVA test with the *p* value of 0.5839.

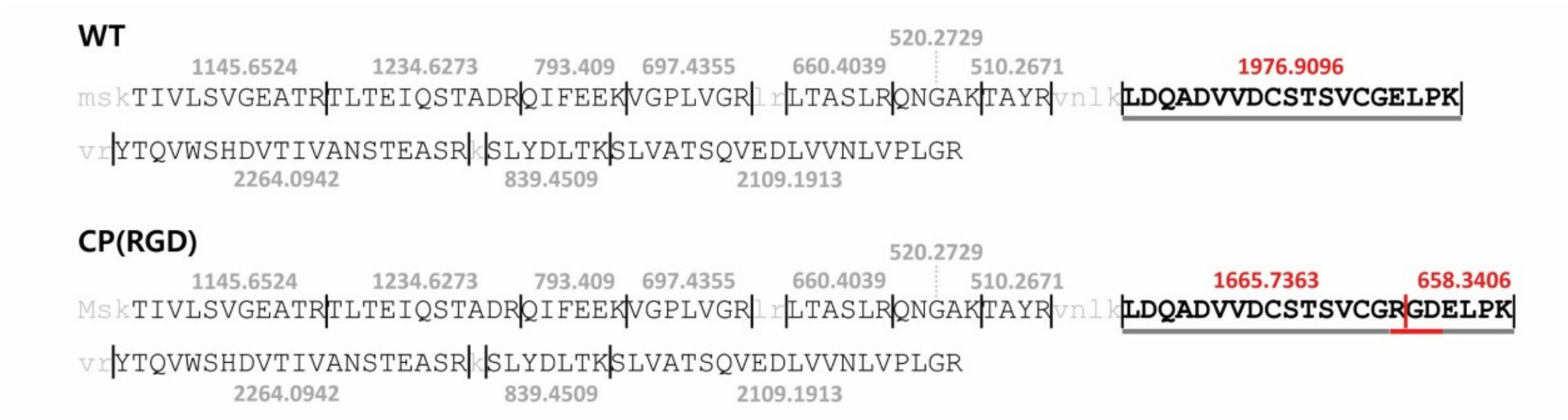

Figure S2. Trypsin cleavage patterns and masses, related to Fig. 5

Amino acid sequence alignment of untagged CP (WT) and RGD-tagged CP [CP(RGD)] shows the trypsin cleavage sites and the calculated masses of the digested fragments. The engineered RGD insertion is shown within the CP(RGD) sequence. Shared peptide masses are in gray and the masses of those unique to WT and CP(RGD) are indicated as bold (red).

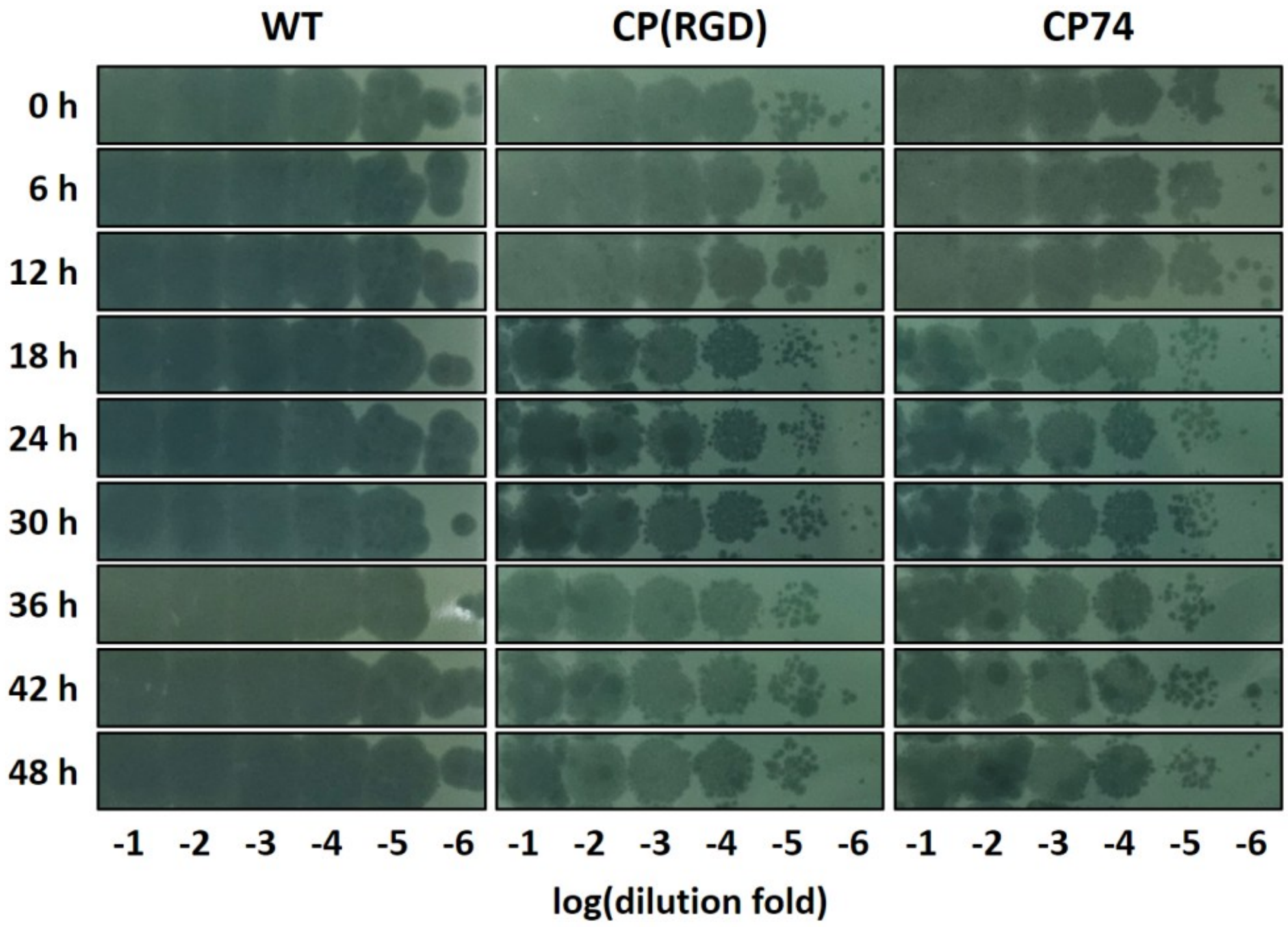

Figure S3. Stability of engineered phage particles in vitro, related to Fig. 6

The in vitro stability of PP7 phages particles carrying WT, CP(RGD), and CP74 [CP(CGRSG)] capsids was assessed by spotting assays. CP74 [CP(CGRSG)] was included as a mock control for CP(RGD). Phage samples in phage buffer (~10^9 pfu/ml) were incubated at 25˚C for 48 h and aliquots were collected at 6-h time intervals as indicated. Each sample was serially diluted and spotted onto lawns of the indicator strain, PAO1ΔRF. Numbers below designate the log(dilution fold) of each sample.

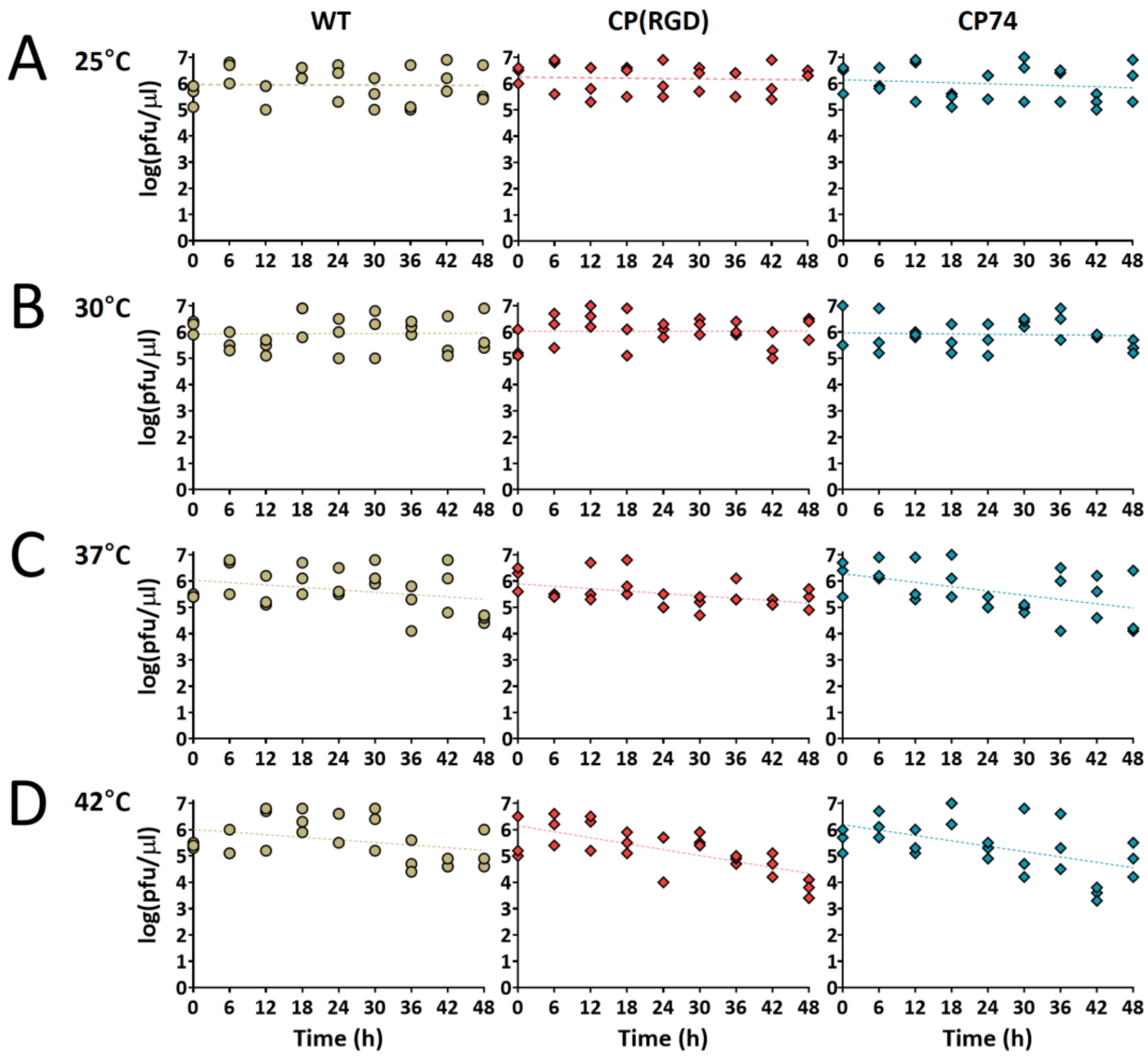

Figure S4. Stability of engineered phage particles at other temperatures, related to Fig. 6

The in vitro stability of PP7 phage particles carrying WT, CP(RGD), or CP74 [CP(CGRSG)] capsids was evaluated by plaque assays at different temperatures. Phage samples prepared in phage buffer (~10^9 pfu/ml) were incubated at 25°C (**A**), 30°C (**B**), 37°C (**C**), or 42°C (**D**) for 48 h, and aliquots were collected at 6-h intervals as indicated. Samples were appropriately diluted, and phage titers were determined using the indicator strain PAO1ΔRF. Phage titers are presented as log(pfu/μl). Data are from three independent biological replicates, with individual data points shown. Dashed lines indicate linear regression fits representing the overall decay trends under each condition; corresponding R^2 values below 0.5 are not shown.

Table S1. Detailed Information of ITRs from MS2 and PP7

Clone ID	Insertion[a]		Titer[b]	No[c]	Clone ID	Insertion[a]		Titer[b]	No[c]
MP23	Asp(GAC^)	C198	1	5	C48	5´-UTR	C48	8	5
MP55	His(C^AC)	C292	5	9	MP15	Gly(GG^A)	G123	7	2
MP285	Gly(G^GT)	G982	1	1	MP18	Leu(CTA^)	A133	6	2
MP340	Gly(GGG^)	G1149	5	3	MP19	Ser(TC^G)	C135	5	5
MP391	Leu(C^TC)	C1300	5	3	MP149	Val(GT^G)	T525	5	1
LP29	Cys(T^GT)	T1796	3	3	MP218	Gly(GG^A)	G732	6	3
RP1	Met(AT^G)	T1762	4	1	MP299	Ala(GC^T)	C975	2	3
RP77	Arg(C^GG)	C1989	2	5	CP73	Cys(TG^C)	G1646	6	2
RP86	Ile(AT^A)	T2017	4	4	CP74	Gly(GGC^)	C1650	6	3
RP126	Gln(C^AA)	C2136	5	6	CP82	Thr(A^CT)	A1672	1	2
RP128	Thr(ACG^)	G2144	4	37	LP11	Lys(AA^G)	A1815	6	3
RP129	Phe(TTC^)	C2147	4	1	LP14	Arg(C^GG)	C1823	7	1
RP136	Gly(G^GG)	G2166	3	1	LP15	Arg(C^GT)	C1826	7	2
RP171	Pro(CC^G)	C2272	3	6	LP18	Arg(C^GT)	G1835	6	3
RP178	Arg(CGC^)	C2294	2	1	LP21	Glu(G^AG)	G1844	6	2
RP190	Gly(GG^G)	G2329	2	1	LP55	Val(GT^G)	T1947	5	3
RP201	Lys(AAA^)	A2363	4	1	RP180	Gly(GGA^)	A2391	3	2
RP277	Pro(C^CT)	C2589	4	1	RP241	Ala(G^CG)	G2572	2	1
RP385	Arg(CGT^)	T2915	3	1	RP289	Val(GTA^)	A2718	8	5
RP389	Val(GT^C)	T2926	5	1	RP291	Gly(GGT^)	T2724	8	10
RP446	Leu(CTC^)	C3098	4	1	RP344	Glu(GA^A)	A2882	7	4
RP455	Pro(CCG^)	G3125	4	4	RP351	Met(A^TG)	A2902	1	2
RP461	Val(GT^A)	T3142	5	1	RP430	Tyr(TAC^)	C3141	2	1
RP464	Lys(AA^G)	A3151	5	1	RP475	Arg(CGC^)	C3276	2	1
RP468	Gly(GGG^)	G3164	5	2	RP488	Leu(CT^G)	T3314	1	2
RP476	Thr(A^CC)	A3186	5	10	RP537	Glu(GAG^)	G3462	6	2
RP478	Gly(G^GT)	G3192	4	3					
RP531	Val(GTT^)	T3353	2	3					
G3483	3´-UTR	G3483	5	12					
Total				128					72

[a]The precise ITR positions are denoted by ^; The numbers represent the 5´-nucleotide of the ITRs.
[b]Titers are expressed as log(pfu/μl).
[c]No represent the redundant numbers of the corresponding clones obtained.

Table S2. Bacterial strains and plasmids used in this study

Strain or plasmid	Relevant characteristics or purpose[a]	Reference or source
Pseudomonas aeruginosa		
PAO1	Wild type laboratory strain for PP7 propagation	Laboratory collection
PAO1ΔRF	PAO1 with in-frame deletions of R and F pyocins	Laboratory collection
PAK	Wild type laboratory strain	Laboratory collection
PAKΔRF	PAK with in-frame deletions of R and F pyocins	Laboratory collection
PAK(PP7)	PAK with the chromosomally integrated PP7 cDNA at *att*Tn*7*; Gm^R	(27)
PAKΔRF(PP7)	PAKΔRF with the chromosomally integrated PP7 cDNA at *att*Tn*7*; Gm^R	This study
Escherichia coli		
DH5α	Multipurpose cloning	Laboratory collection
HB101	Cloning and plasmid propagation host	Laboratory collection
SM10(λ*pir*)	Conjugative donor strain with RP4 functions with λpir^+	Laboratory collection
C3000	Host strain for MS2 propagation	Laboratory collection
HB101(MS2)	HB101 with the chromosomally integrated MS2 cDNA at *att*Tn*7*; Gm^R	This study
Phage		
PP7	*Pseudomonas* phage *Pepevirus rubrum*	Laboratory collection
MS2	*Escherichia coli* phage *Emesvirus zinderi*	Laboratory collection
CP73	PP7 with 15 bp scar at CP73 ITR (i.e., between G1646 and C1647)	This study
CP74	PP7 with 15 bp scar at CP74 ITR (i.e., between C1650 and G1651)	This study
CP82	PP7 with 15 bp scar at CP82 ITR (i.e., between A1672 and C1673)	This study
CP(RGD)	PP7 with 9 bp insertion for RGD tagging at CP74 ITR (i.e., between C1650 and G1651)	This study
MP(RGD)	PP7 with 12 bp insertion for RGD tagging at MP149 ITR (i.e., between T525 and G526)	This study
Plasmids		
pUC18-mini-Tn*7*T	mini-Tn*7*-based chromosomal integration vector; Cb^R, Gm^R	(27)

mTn-PP7	pUCP18-mini-Tn7T with PP7 cDNA fused to the T7 promoter; Cb^R, Gm^R	(10)
mTn-MS2	pUCP18-mini-Tn7T with MS2 cDNA fused to the T7 promoter; Cb^R, Gm^R	(10)
mTn-CP73	mTn-PP7 with 15 bp at CP73 ITR (i.e., between G1646 and C1647); Cb^R, Gm^R	This study
mTn-CP74	mTn-PP7 with 15 bp at CP74 ITR (i.e., between C1650 and G1651); Cb^R, Gm^R	This study
mTn-CP82	mTn-PP7 with 15 bp at CP82 ITR (i.e., between A1672 and C1673); Cb^R, Gm^R	This study
mTn-CP(RGD)	mTn-PP7 with 9 bp insertion for RGD tagging at CP74 ITR (i.e., between C1650 and G1651); Cb^R, Gm^R	This study
mTn-MP(RGD)	mTn-PP7 with 12 bp insertion for RGD tagging at MP149 ITR (i.e., between T525 and G526); Cb^R, Gm^R	This study
pJN105	Arabinose-inducible expression vector with P_{BAD} promoter; Gm^R	
pJN-RP_{MS2}	pJN105 expressing MS2 RNA-dependent RNA polymerase; Gm^R	This study
pJN-RP_{PP7}	pJN105 expressing PP7 RNA-dependent RNA polymerase; Gm^R	This study
pTNS2	miniTn*7* transposition helper plasmid (*tnsABCD*); Cb^R	(27)
pRK2013	Conjugative helper plasmid with RP4 functions; Km^R	(27)

[a]Gm^R, gentamicin-resistant; Cb^R, carbenicillin- and ampicillin-resistant; Km^R, kanamycin-resistant.

Table S3. Primers used in this study

Primer or probe	Relevant characteristics or purpose	Oligonucleotide sequence (5'–3')[a]
PP7-F	PCR for full-length PP7 cDNA synthesis	AC**ACTAGT**TTATTAATACGACTCAC (SpeI)
PP7-R	PCR for full-length PP7 cDNA synthesis	GC**AAGCTT**TGGTCGAAGTCCGCTGAG (HindIII)
NotI-mini	PCR for 15-bp scar at ITRs verification	TGCGGCCGCA
mTn7-PP7-F	SOEing PCR for RGD insertion PP7 construction	CAGGAATTCCTCGAGAAGCTGCGGCCGCTTAATACGACTCACT
mTn7-PP7-R	SOEing PCR for RGD insertion PP7 construction	CGAGGTACCGGGCCCAAGCTCCCGGGTGGTCGAAGTCCGC
mTn-CP74(RGD)-F	SOEing PCR for CP74(RGD) construction	GGCCGTGGTGATGAGCTTCCGAAAG
mTn-CP74(RGD)-R	SOEing PCR for CP74(RGD) construction	CTCATCACCACGGCCGCAGACG
mTn-MP149(RGD)-F	SOEing PCR for MP149(RGD) construction	GTGAGAGGTGACGTGAACCTCCCTATGC
mTn-MP149(RGD)-R	SOEing PCR for MP149(RGD) construction	CACGTCACCTCTCACCAGACTCTTATTC
rpoA-RT-F	RT-qPCR for *rpoA*	CATCGGTGACCTGATCCAGCG
rpoA-RT-R	RT-qPCR for *rpoA*	ACCACGGGAAGCCAGAACGTC
PP7-RT-F1	RT-qPCR for PP7 at MP region	ATGTCCAAAACCATCGTTCTTTCGGTCGGCGAGGC
PP7-RT-R1	RT-qPCR for PP7 at MP region	GAGAACGGTTCTACCTTCCG
PP7-RT-F2	RT-qPCR for PP7 at RP region	ATGGAAACCCTTAGTAGCGC
PP7-RT-R2	RT-qPCR for PP7 at RP region	GAGAACGGTTCTACCTTCCG
Tn7L	PCR for mini-Tn*7* insertion at *att*Tn*7* verification	ATTAGCTTACGACGCTACACCC
glmS-UP	PCR for mini-Tn*7* insertion at *att*Tn*7* verification	CTGTGCGACTGCTGGAGCTGA

[a]Underlining denotes the engineered restriction enzyme sites; the mutated nucleotide residues are boldfaced and underlined
